# More efficient formation of longer-term representations for word forms at birth can be linked to better language skills at 2 years

**DOI:** 10.1016/j.dcn.2022.101113

**Published:** 2022-05-13

**Authors:** Emma Suppanen, István Winkler, Teija Kujala, Sari Ylinen

**Affiliations:** a Cognitive Brain Research Unit, Department of Psychology and Logopedics, Faculty of Medicine, University of Helsinki, Helsinki, Finland; b Institute of Cognitive Neuroscience and Psychology, Research Centre for Natural Sciences, Hungarian Academy of Sciences, Hungary; c Logopedics, Welfare Sciences, Faculty of Social Sciences, Tampere University, Finland

**Keywords:** Auditory processing, Electroencephalography (EEG), Event-related potential (ERP), Predictive coding, Language development

## Abstract

Infants are able to extract words from speech early in life. Here we show that the quality of forming longer-term representations for word forms at birth predicts expressive language ability at the age of two years. Seventy-five neonates were familiarized with two spoken disyllabic pseudowords. We then tested whether the neonate brain predicts the second syllable from the first one by presenting a familiarized pseudoword frequently, and occasionally violating the learned syllable combination by different rare pseudowords. Distinct brain responses were elicited by predicted and unpredicted word endings, suggesting that the neonates had learned the familiarized pseudowords. The difference between responses to predicted and unpredicted pseudowords indexing the quality of word-form learning during familiarization significantly correlated with expressive language scores (the mean length of utterance) at 24 months in the same infant. These findings suggest that 1) neonates can memorize disyllabic words so that a learned first syllable generates predictions for the word ending, and 2) early individual differences in the quality of word-form learning correlate with language skills. This relationship helps early identification of infants at risk for language impairment.

## 1 Introduction

Learning to communicate by speech is a major developmental achievement for human infants. Although they appear to grasp speech quickly and spontaneously, it is one of the most complex tasks faced during infancy (e.g., Kuhl, 2004; Werker and Curtin, 2005). However, there is considerable variation in children’s language development (Fenson et al., 1994). For example, Fenson and colleagues (1994) found that the number of words produced at 2 years ranged from 7 to 668. Learning effects tend to accumulate, and it is therefore crucial to support those with poorer language skills or delayed language development as early as possible. Since early diagnostics is an essential part of remediation (Sigman et al., 2014), we need to develop tools for detecting infants at risk for poor language skills or language impairments before we can measure the actual language outcomes from behavior. The present study aimed to find a neural index of later language ability, which can be safely measured from the brain activity of newborn infants with electroencephalography (EEG).

To this end, we measured neural responses reflecting word-form learning, i.e., learning syllable or morpheme combinations, in newborns. We used a stimulus paradigm utilizing predictive processing, because predictive processes have been shown to play an important role in language functions (Pickering and Garrod, 2013, Kuperberg and Jaeger, 2016). These processes are used to recognize words (Gagnepain, 2012) and predict upcoming speech sounds based on the rules of the native language (DeLong et al., 2005, Ylinen et al., 2016). Further, predictive information has been found to increase the perceived clarity of speech when acoustic clarity is degraded (Wild et al., 2012) and to decrease reaction times to words (for a review, see Kuperberg and Jaeger, 2016). In addition, infants appear to utilize predictive inference from birth (Ruusuvirta et al., 2009, Näätänen et al., 2010, Trainor, 2012, Háden et al., 2015), and they extract regularities from speech-like sequences (Teinonen et al., 2009, François et al., 2017, Suppanen et al., 2019). Linking infant predictive processing with language development, Ylinen et al. (2017) have demonstrated that predictability facilitates word recognition and learning in 12-month-old infants.

One consequence of learning is that one can infer to the whole from detecting a part, as it generally occurs in everyday perception (Gregory, 1980). Because predictive processing is functional at birth and the infant brain arguably uses it in speech processing, we hypothesized that the precision of neonates’ predictive processing for familiarized pseudowords varies as a function of their learning ability. We extracted a measure of predictive precision from electric neonatal brain responses utilizing the infant equivalent of auditory mismatch negativity (MMN, Näätänen et al., 1978; for a recent review, see Fitzgerald and Todd, 2020), the mismatch response (MMR^1^). MMN/MMR is a prediction error signal (Winkler et al., 1996; for reviews see Garrido et al., 2009; Bendixen et al., 2012) elicited by infrequent “deviant” sounds violating some regularity of the preceding stimulus sequence, such as a repeating “standard” sound. MMN is not only elicited by violating acoustic regularities extracted from the immediately preceding sequence, since prediction violations detected by MMN can be based on longer-term effects of learning, such as learning of phoneme representations (e.g. Winkler et al., 1999; Ylinen et al., 2010; for a review, see Näätänen et al., 2001). For the infantile MMR, Ylinen and colleagues (2017) showed that an unexpected word ending elicits a word-level prediction error MMR, because hearing the beginning of a familiar word generates a prediction for its ending. We employed this effect for exploring word-form learning that leads to longer-term representations in sleeping infants and tested whether a neural measure of the quality of word-form learning could predict later language outcomes. We hypothesized that its variation across neonates will positively correlate with measures of the infants’ subsequent language development. Language outcome was assessed by measuring expressive language ability at 24 months of age.

## 2 Methods

### 2.1 Participants

75 healthy, full-term newborn infants (see Table 1) born into Finnish-speaking families participated in the study. The study protocol was approved by the Ethics Committee for Gynecology and Obstetrics, Pediatrics and Psychiatry of the Hospital District of Helsinki and Uusimaa, and EEG measurements were conducted in Jorvi hospital in Espoo, Finland in compliance with the Declaration of Helsinki. Written informed consent was obtained from at least one of the parents of each participant prior to the experiment. Data of 11 infants were excluded due to technical problems, or not reaching the criterion of 50 accepted epochs per stimulus type, resulting in the inclusion of 64 infants in the study.

**Table 1:** Description of the participants (mean, and standard deviation [SD]).

	All participants		Included participants		Participants included in correlation analysis	
Variable	Mean	SD	Mean	SD	Mean	SD
N (male/female)	75 (41/34)	N/A	64 (31/33)		45 (22/23)	N/A
Gestational age (weeks)	40.1	0.9	40.1	0.9	40.2	1.0
Age at measurement (days)	9.3	4.4	9.0	4.4	9.0	4.6
Birth weight (g)	3530	423	3532	428	3555	404
5-min Apgar score (range)	7–10		7–10		7–10	

### 2.2 Stimuli

Stimuli were recorded in a sound-isolated studio (microphone: Rode NT2-A, Rode Microphones, Sydney, Australia; audio interface: Digidesign Digi 002, software: Pro Tools 12, both: Avid Audio, Berkeley, CA, USA) where a female and a male native Finnish speaker repeated (pseudo)words several times. The stimulus material included pseudowords /’kut:o/, /’tek:ɑ/, /’tet:o/, /’kup:e/ and a word /’kuk:ɑ/. All of these are legal with respect to the Finnish phonology. Syllables were isolated from their context and assessed by two native Finnish speakers. From both the male and the female speaker, for each syllable, the two most prototypical exemplars without clear co-articulatory cues revealing the original context were chosen for the experiment. The syllables were then processed with Praat (Boersma and Weenink, 2010): durations and intensities were equalized as closely as possible, separately for the stressed and unstressed positions. The (pseudo)words presented in the study were constructed by pairing the exemplars of the first-position (stressed) syllables with the exemplars of the second-position (unstressed) syllables in all possible combinations, separately for the male and female speaker (4 combinations/(pseudo)word/speaker).

The total duration of the (pseudo)words was 426 ms: the first syllable was ≈ 90 ms, a silent pause mimicking the occlusion phase of a stop consonant was ≈ 210 ms, and the second syllable was ≈ 126 ms. Thus, the second syllable onset was at ≈ 300 ms from the onset of the (pseudo)word. The interstimulus interval (offset to onset) was ≈ 510 ms. The stimuli were presented with Presentation 17.2. Software (Neurobehavioral Systems Ltd., Berkeley, CA; USA) via a Genelec speaker placed approximately 40 cm from the infant’s head directly in front of the infant. The approximate sound level was 65 dB sound pressure level (SPL).

### 2.3 Study design

The experiment consisted of two phases, the familiarization phase and the test phase. In the familiarization phase pseudowords /’kut:o/ and /’tek:ɑ/ (referred as *AB* and *CD*, respectively, where *A*, *B*, *C*, and *D* represent different syllables) were presented (p = 0.5) in three stimulus blocks, each containing 250 stimuli and lasting for 11.7 min, altogether. We expected the infants to form an internal model of the pseudowords presented in the familiarization phase so that familiar initial syllables would evoke predictions for the syllable to follow them (i.e., *A* evokes the prediction of *B*, whereas *C* evokes the prediction of *D*). 80% of the stimuli were spoken by one speaker, and the remaining by the other speaker. Infants either heard only predominantly male-speaker or only predominantly female-speaker blocks. The test phase was presented in four oddball blocks, each containing 750 stimuli, 46.8 min overall. The order of the stimuli was pseudo-randomized so that a deviant was followed by at least two standards and each block started always with at least eight standards. The blocks were homogenous with respect to the gender of the speaker and each infant heard either male-only or female-only blocks. The speaker gender variables were fully crossed both in the familiarization and the test phase in order to balance the possible effects of the speaker’s gender, resulting in four groups of participants (average 19 participants in each). The four groups were collapsed in the current data analysis (the effects of speaker gender will be assessed in a separate study).

**Fig. 1:**
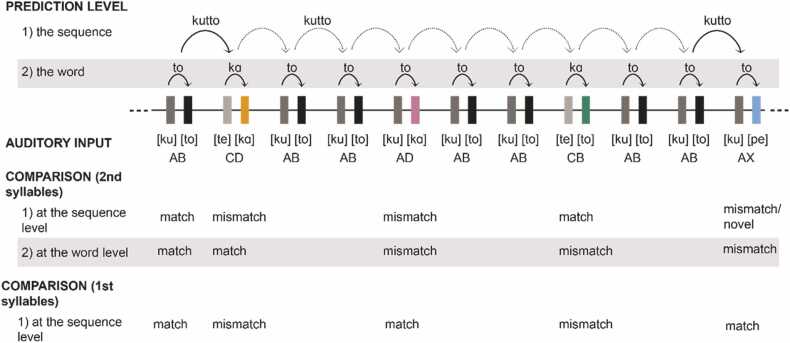
An example of stimulus sequences. *AB* and *CD* were introduced in the familiarization phase, and familiar word beginnings were expected to generate predictions of the endings. Note that a deviant was followed by at least two (in most cases more) standards.

In the test phase, an oddball paradigm with repetitive standard and occasional deviants was used to probe prediction and learning effects (see Fig. 1). The familiar pseudoword *AB* (/’kut:o/; p = 0.8) served as the frequent standard stimulus in an oddball sequence with four kinds of rare deviants: *AD*, *AX* (*X* denoting a novel syllable), *CD*, and *CB* (p = 0.05 for each). 1) the other familiarized pseudoword *CD* (/’tek:ɑ/; familiar pseudoword deviant), 2) a combination of two familiar syllables, forming a non-familiarized pseudoword *CB* (/’tet:o/), 3) the other combination of familiar syllables forming a non-familiarized word *AD* (/kuk:ɑ/; a flower), and 4) a pseudoword starting out as the standard but ending with a novel (not familiarized) second syllable *AX* (/kup:e/).

For *AD* and *AX*, syllables *D* and *X* were hypothesized to elicit two overlapping MMRs because 1) they violated the local sequence-level regularity extracted from the preceding sequence and 2) the word-level prediction learned during the familiarization phase (Ylinen et al., 2017) and thus induced by the first syllable (*A* -> *B*). The response to the novel (not familiarized) syllable *X* was also expected to evoke a novelty response (Kushnerenko et al., 2013). Thus, comparing the responses between *AD* and *AX* may reflect the effects of syllable novelty. Both *D* and *X* following *A* differ from the local standard as well as from the familiarized *AB*, with *D* having been familiarized as part of the *CD* pseudoword, while *X* not having appeared previously.

For *CD* and *CB*, the initial syllable *C* was expected to elicit the sequence-level MMR since it breaks the repetition of *AB*. The final syllables, in turn, were hypothesized to evoke different responses for the familiarized pseudoword *CD* and the unfamiliar pseudoword *CB*. This is because unlike *D*, *B* violated the learned *C* -> *D* prediction and should elicit word-level prediction error (Ylinen et al., 2017). Thus, the difference between the responses to the second syllables of *CD* and *CB* reflects how well the neonates had learned to expect *D* after hearing *C* and provides an index for the quality (precision) of forming longer-term representations for word forms during the familiarization phase. Further, both *B* and *D* appeared as the second syllable of a familiarized pseudoword with *B* matching the second syllable of the standard (*AB*) stimulus of the oddball sequence. Therefore, difference between the responses to these syllables cannot be due to “syllable learning” (i.e., mere syllable familiarity). In sum, learning the regularity of the actual sequence should be reflected in all deviant responses while longer-term word-learning should affect all but the response to *D of* the *CD* deviant (See Fig. 1). Therefore, comparison of the responses to *CB* and *CD* is the optimal measure separating the effects of longer-term learning from 1) local sequence learning, 2) syllable learning, and 3) learning the position of the syllable within the pseudowords.

### 2.4 Data collection

Infants were sleeping on their back in a crib while EEG was recorded with 500 Hz sampling rate, from DC to 100 Hz with 18 active electrodes (headcap: ActiCap; amplifier: BrainProducts QuickAmp 10.08.14; software BrainVision Recorder 1.20.0801; all: Brain Products GmbH, Gilching, Germany) placed according to the international 10/20 system at Fp1/2, F7/8, F3/4, Fz, C3/4, Cz, P7/8, P3/4, Pz, Oz) and over the mastoids (LM and RM). The data were referenced to the average of all electrodes during the recording.

When the infants reached the age of 24 months, their parents filled out the Finnish version (Lyytinen, 1999) of the MacArthur-Bates communicative development inventory (CDI; Fenson et al., 1994). 56 of the 75 families (75%) provided this information.

### 2.5 Data analysis

We only report here the data collected in the test phase. EEG signals were pre-processed with BESA Research 6.0 (Besa GmbH, Gräfelting, Germany) and MATLAB Release 2018b (The MathWorks, Inc., Natick, Massachusetts, USA), using EEGlab 2019.0 (Delorme and Makeig, 2004) and in-house Matlab scripts (CBRUPlugin2.1b, Tommi Makkonen, Cognitive Brain Research Unit, University of Helsinki). First, the signals were bandpass-filtered offline (high-pass 0.5 Hz, low-pass 30 Hz, slope 24 dB/octave) and segmented into − 100–800 ms epochs with respect to word onset, separately for each stimulus and participant. The responses to the two standard stimuli immediately following a deviant and epochs with their amplitude exceeding ± 80 µV were excluded from the analysis. After artefact rejection, the average number of remaining responses to the standards was 992 (SD = 126), while that to the deviants was 128 (SD = 17), separately for each deviant. The signals were then re-referenced to the average of the two mastoid electrodes.

Epochs were averaged separately for each stimulus type. The baseline was corrected by the average voltage within time-windows immediately preceding the divergence point of the deviants. The deviants *AD* and *AX* diverged from the standard at 300 ms and any difference in the responses to their identical first syllables before this time point must have been due noise. Therefore, the responses were baseline-corrected by the average voltage in the 300 ms interval before the onset of the second syllable to compensate the effect of the pre-stimulus-divergence noise on the response amplitude difference. The deviants *CD* and *CB* started with a different initial syllable than the standard. They were baseline-corrected by the 100 ms pre-stimulus interval. The averaged response to the standard stimulus (baseline-corrected the same way as the corresponding deviant) was subtracted from those to the deviants in order to quantify the deviance-related responses. Two time-windows (time window 1: 200 – 300 ms, and time window 2: 550 – 650 ms) were selected for statistical analysis based on previous literature (especially Ylinen et al., 2017) and considering the onset of the second syllable. Time window 1 was only employed for deviants *CD* and *CB*, because *AD* and *AX* are identical to the standard in this period, which is fully covered by the baseline for these two deviants.

Statistical analysis was carried out with SPSS 25 (IBM, Armonk, New York, US). Normality of the data distributions was tested using the Shapiro-Wilk test (W). To test whether the deviance related responses were statistically significant (*α* = .05), the mean voltages, measured for the average of the three central and three frontal electrode locations (six electrode locations, altogether) from the selected time windows, were submitted to one sample Student’s *t*-tests (i.e., comparing them to zero). Effect sizes are reported as Cohen’s *d*. Repeated measures ANOVAs (2 ×2×3 levels) were conducted on the difference amplitudes separately for each time window and first syllable (i.e. time window 1 testing was only done for deviants starting with the *C* syllable, while time window 2 testing was done both for deviants starting with *A* and *C*, but separately, because the baselines differ between them). The ANOVA factors were *Deviant* (*CD* vs. *CB* or *AD* vs. *AX*), *Frontality* (F vs. C line) and *Laterality* (left vs. middle vs. right [electrodes F/C 3, z, and 4]). We will only report the effects involving the *Deviant* factor in Results, as only those are related to our hypotheses; the full set of results are shown in Tables A.1, A.2, and A.3. When sphericity could not be assumed, Greenhouse-Geisser correction was applied where applicable and the ε correction factor is reported. Significant (α = .05) effects were followed up by Bonferroni-corrected pairwise comparisons. Cohen’s *d* values were calculated for each pairwise comparison.

Given that the amplitude of the prediction error response is assumed to reflect the specificity of the violated prediction (Friston, 2005, Winkler, 2007), the difference between the electrical brain response amplitudes elicited by predicted and unpredicted word endings was used for testing the association between longer-term learning effects at birth (see Section 2.3) and later language ability (Ylinen et al., 2017). The mean fronto-central amplitude for *CB* (the average of six locations along the F and C lines) was subtracted from that for *CD* in time window 2. (Note that the direction of this subtraction is arbitrary as there is no external reference for the difference between two responses.) This variable was then rank correlated with the mean length of the three longest utterances (MLU, or M3L, taken from the CDI) at 24 months. MLU is a widely used index of expressive language ability, tapping both lexical and morphosyntactic proficiency (see Scarborough, 1990; DeThorne et al., 2005, Rice et al., 2006; Kunnari et al., 2012; Jalilevand and Ebrahimipour, 2014). We used this measure in line with both Bettoni et al. (2020), who found infants’ rule-based learning ability to predict MLU outcome, and Mittag et al. (2021), who measured infants’ neural activity and assessed their linguistic and non-linguistic communication skills. Among different measures, they found MLU/M3L to correlate most consistently with neural activity patterns, suggesting that it may be a particularly useful measure for the current neurocognitive setup. 45 infants had all required measures and could be included in this analysis.

## 3 Results

**Fig. 2:**
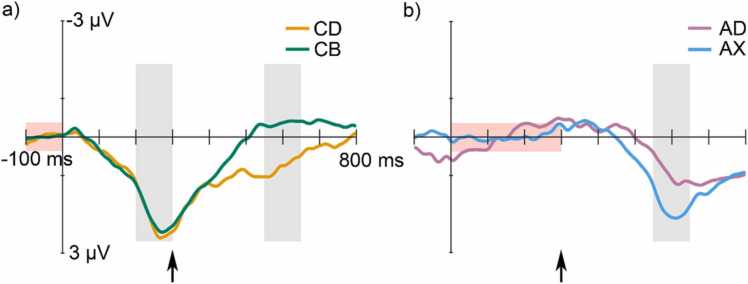
Group-averaged (N = 64) frontal (electrode Fz) deviant-minus-standard difference responses. (a) The deviants *CD* (/tek:ɑ/, the familiarized pseudoword) and *CB* (/tet:o/, an unfamiliar pseudoword consisting of the combination of the two familiar syllables). (b) The deviants *AD* (/kuk:ɑ/, an unfamiliar word consisting of the combination of two familiar syllables) and *AX* (/kup:e/, a novel pseudoword starting out as the standard, but ending with a syllable that was not familiarized). The time windows (200 – 300 ms and 550 – 650 ms) used for measuring the deviant-related responses are marked with light gray vertical rectangles, and the baselines used for baseline correction with pink horizontal rectangles. The y-axis is at 0 ms (onset of the first syllable) and the onset of the second syllable is marked by a black arrow. The significant (see Table 2) deviant responses showed that the newborns were able to differentiate each of the four different deviants from the standard. The difference between the responses to *CB* and *CD* in the second time window served as the measure of the quality of formation of longer-term representations for word forms.

**Fig. 3:**
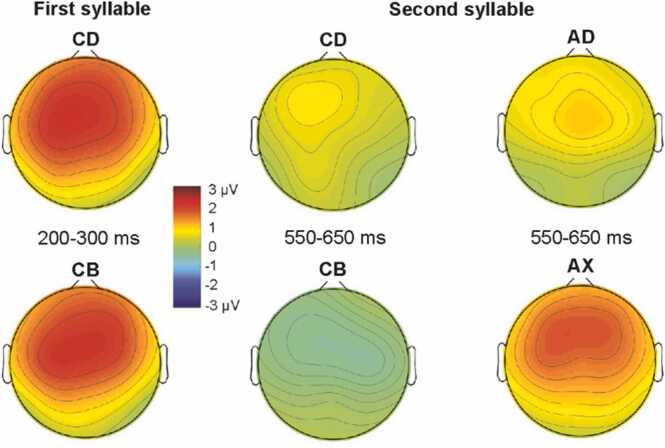
The scalp distributions for the group-averaged (N = 64) neural activity (mean neural response amplitudes) in Time window 1 corresponding to the first syllable (*CD* and *CB*, only) and in Time window 2 corresponding to the second syllable. These distributions are consistent with those previously observed for the MMR, indicating summed activity from the auditory brain areas (for review see Kushnerenko et al., 2013).

All deviant stimuli elicited a response significantly differing from the response to the standard (see Fig. 2, Fig. 3 as well as Figure A.1; Table 2 for the amplitudes and *t*-test results) suggesting that the infants’ brain could distinguish the rare stimuli from the frequent one.

**Table 2:** Group-averaged (N = 64) deviant-minus-standard difference amplitudes in the two time windows (time window 1 only for the *CD* and CB deviants, because *AD* and *AX* do not yet deviate from AB at this latency) together with the results of the one-sample t-tests, separately for each deviant. Mean amplitudes (in bold) and standard deviations (in parentheses) are given¬ for the average of the six selected frontal and central channels. One-sample t-statistics (t, df – degrees of freedom, in parentheses, p – significance level, Cohen’s d – effect size) are also shown.

Difference response	*CD* /tek:ɑ/	*CB* /tet:o/	*AD* /kuk:ɑ/	*AX* /kup:e/
Time window 1 (200 – 300 ms)	**2.00** (1.4) *t*(63) = 11.19, *p* = .001, *d* = 1.4	**1.98** (1.8) *t*(63) = 8.86, *p* = .001, *d* = 1.11	N/A	N/A
Time window 2 (550 – 650 ms)	**.51** (1.4) *t*(63) = 2.97, *p* = .004, *d* = 0.37	**-.38** (1.4) *t*(63) = −2.16, *p* = .034, *d* = −0.27	**.66** (1.4) *t*(63) = 3.81, *p* = .001, *d* = 0.47	**1.37** (1.7) *t*(63) = 6.61, *p* = .001, *d* = 0.83

Repeated measures ANOVAs [*Deviant* (*CD* vs. *CB* and, separately, *AD* vs. *AX*) × *Frontality* (Frontal vs Central) × *Laterality* (left vs. middle vs. right)] for the amplitude measures from time window 1 (200 – 300 ms) did not yield any significant effect involving the *Deviant* factor. However, in time window 2 (550 – 650 ms), for *CD* and *CB* there was a significant main effect of *Deviant* [F(1, 63) = 14.5, *p* = .001, η^2^ = 0.19], with the response to *CB* being significantly more negative than that to *CD* [*p* = .001, *d* = 0.48]. For *AD* and *AX*, there was also a significant main effect of *Deviant* [F(1,63) = 5.98, *p* = .017, η^2^ = 0.09] and a significant *Deviant* × *Frontality* × *Laterality* interaction [F(2, 126) = 4.86, *p* = .021, ε = 0.659, η^2^ = 0.07]. According to Bonferroni-corrected pairwise comparisons, the deviant *AX* elicited a significantly more positive response than *AD* (*p* = .017, *d* = 0.31), at the Fz [*p* = .038, *d* = 0.27], F4 [*p* = .002, *d* = 0.41], C3 [*p* = .018, *d* = 0.21] and C4 [*p* = .033, *d* = 0.27]. For the full ANOVA results, see Tables A.1, A.2, and A.3. Rank correlations between the three contrasts (local deviance: *AB* vs *AD*; syllable novelty: *AD* vs. *AX*; word-form learning: *CD* vs *CB*) showed that while word-form learning is likely separate from sequential deviance and syllable learning (non-significant correlation), the other two are interrelated (significantly correlated; Table A.4).

**Fig. 4:**
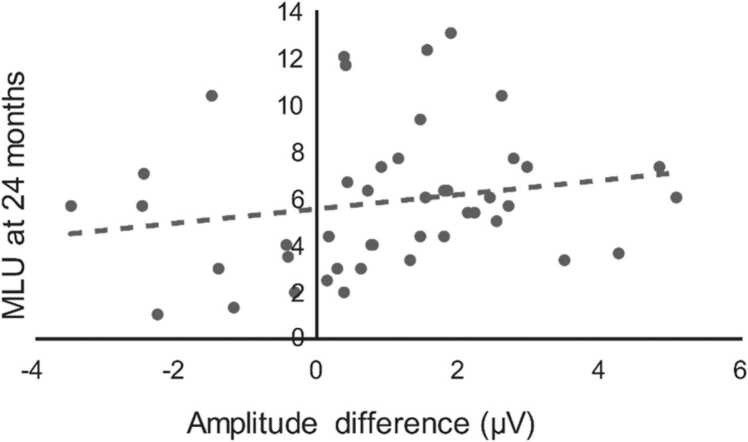
Regression (N = 45, see Methods) between the neonatal word-learning effect reflected by the fronto-central *CD*-minus-*CB* brain response amplitude difference and the mean length of utterance (MLU) at 24 months. The positive correlation means that larger word-learning effect at birth is associated with a higher expressive vocabulary at 24 months.

Correlation analysis showed that the learning effect operationalized by the amplitude difference of word-level prediction error in time window 2 (*CB*-minus-*CD*; average of the six frontal and central electrodes) significantly rank correlated with the mean length of utterance measured at 24 months of age (ρ = 0.33, *p* = .025, n = 45). The trend line for the significant correlation is shown in Fig. 4. Rank correlations between MLU and the simple local deviance effect (*AD* vs. *AB*), between MLU and the novelty effect (*AD* vs. *AX*), or between the amplitude difference of word-level prediction error and CDI vocabulary measure were not significant (Tables A.5 and A.6, respectively).

## 4 Discussion

The current study investigated whether forming longer-term representations for word forms from speech exposure at birth predicts later language ability. Word-form learning was assessed by measuring ERP responses to (pseudo)words starting with a syllable identical to a previously familiarized pseudoword but ending with a different syllable. If learned, the first syllable of the familiarized pseudowords was expected to induce predictions for their endings. Violating these predictions should elicit a word-level prediction error response in the brain (Ylinen et al., 2017). The amplitude of the prediction error response is assumed to reflect the specificity of the violated prediction (Friston, 2005, Winkler, 2007). Therefore, the difference between the brain’s response to predicted and unpredicted endings can be used to assess the quality of longer-term representations formed for word forms (i.e., learning the word forms during the familiarization phase). We found that this measure significantly correlated with the mean length of the three longest utterances (MLU) assessed through the CDI at 24 months. MLU is a proxy of expressive language ability (DeThorne et al., 2005, Rice et al., 2006, Kunnari et al., 2012, Jalilevand and Ebrahimipour, 2014).

As expected, all four rare deviants elicited significant MMRs, which suggests that the infants distinguished them from the standard and detected that they violated the repetition of the standard. The pattern of the responses suggests that the first time window (200 – 300 ms) most likely reflects the “classical” sequence-related MMR to the acoustic change of the first syllable in *CD* and *CB* deviants (e.g. Háden et al., 2016). However, the pattern of results obtained for the second time window (550 – 650 ms) stems from three sources. 1) Violating the sequential regularity (*AD* and *AX*) could have evoked the “classical” sequential MMR by their second syllables being acoustically different from the second syllable of the repeating standard. Their responses in the second time window show the same (positive) polarity as the response to the acoustic sequence-related deviance observed for *CB* and *CD* in the first time window (see Fig. 1). 2) *AX* elicited a more positive response than *AD*, likely due to the novelty response (Kushnerenko et al., 2013), as *X* was the only syllable in the test sequence to which infants were not previously familiarized. This interpretation is also supported by the wider scalp distribution shown by the response to *AX* than *AD* (Fig. 3). 3) Violating word-level prediction by the second syllable (*AD*, *AX*, and *CB*) probably elicited a prediction error response (Gagnepain, 2012, Ylinen et al., 2017). Within the current stimulus paradigm, this can only be proven for *CB*, the response to which differed significantly from that to the standard *AB* in time window 2 despite both ending with the same second syllable. The difference between *CB* and *AB* shows up as a negative displacement, which suggests that after a positive sequence-level MMR, word-level prediction violations elicit a negative-polarity response (see Fig. 2). Note that, the word-level prediction error for *CB* can only be based on word-form representations formed during familiarization, because if participants did not learn to expect *D* after hearing *C*, the response for *CB* should not have diverged from that for frequent stimulus *AB* in time window 2*.*

Since we assumed that the precision of the violated prediction modulates the resulting prediction error (Friston, 2005, Winkler, 2007), the difference between the responses to deviants *CB* and *CD* in the second time window was expected to reflect longer-term word-form representations resulting from learning during familiarization. These representations generate word-level predictions (Ylinen et al., 2017). The current proxy measure was uncontaminated by syllable and positional learning, because both *D* and *B* were familiar syllables appearing only as the second syllable of the pseudowords both during the familiarization and in the test sequences. The across-infants variation in the size of the *CD* vs. *CB* difference should then index the quality of the longer-term effects of word-form learning in infants. These assumptions allowed us to test whether neonatal capabilities for forming longer-term representations for word forms are related to later expressive language skills. Our word-level prediction-error based measure of longer-term learning effects in neonates significantly correlated with the mean length of utterance at 24 months. Further, no significant correlation was found between MLU and measures of local sequence-learning or between MLU and the effects of syllable novelty. Importantly, this suggests that within the current paradigm, only the longer-term effects of learning syllable or morpheme combinations, but not familiarity/novelty or local sequential effects, contribute to language proficiency at later stages of development.

Our results in newborns are in line with those of Ylinen et al. (2017) who looked at correlation between prediction error responses and language skills cross-sectionally at 12 months and found a strong correlation between prediction error and receptive vocabulary. (Note, however, that in the current study, the neonatal word-level prediction errors correlated with MLU, rather than vocabulary; for similar results, see Bettoni et al., 2020; Mittag et al., 2021.) The current results support Ylinen et al. (2017) earlier findings by demonstrating link between prediction error responses and language outcome in a larger number of participants. Importantly, our current results also add a critical longitudinal aspect to these findings. Since we studied newborn infants, our findings also corroborate recent suggestions that predictive inference facilitates even the earliest learning of word forms (Ylinen et al., 2017, Havron et al., 2019).

In addition to finding the link between longer-term learning effects and later language ability, also the high correlation between sequential MMR (*AD* vs. *AB*) and syllable novelty (*AD* vs. *AX*) is an interesting finding of the current study. While further studies are needed to specify this relationship, this result suggests that general familiarity modulates the response to prediction errors based on locally extracted regularities (see, e.g., Jacobsen et al., 2005, Jacobsen et al., 2021), which is different from the effects of violating previously learned contingencies (Ylinen et al., 2017).

So far, the results have been discussed from the perspective of word-form learning, but in fact the learning of *AB* and *CD* patterns could as well tap morphosyntactic learning, such as learning the combination of different morphemes in a syntactic structure (e.g. *AB* jump+ing, *CD* child+ren). These patterns may also tap some forms of syntactic learning (e.g. *AB* I am, *CD* you are), although artificial grammar studies typically use more complex syllable combinations (e.g. *ABB* vs. *ABC*; see Gervain et al., 2008). In a similar vein, our measure of language ability (MLU) includes both lexical and morphosyntactic components (DeThorne et al., 2005). Thus, our results are not necessarily restricted to word-form learning but may reflect the ability to learn sequences or sequence-based dependencies at different levels of language.

The current results demonstrate that the quality of learning word forms (or other kinds of dependencies) from speech input at birth is an important determinant of later expressive language ability. Based on these findings, we argue that brain responses for word-level predictions can be used as a neonatal index of subsequent language skills. This has important implications for very early detection and remediation of infants at risk for language impairment. To determine the role of our learning index as a potential indicator of language impairments, further research should target infants with familial risk for various language impairments.

## 5 Conclusions

In conclusion, we found that forming longer-term representations for word forms at birth predicts expressive language skills at 24 months. We showed that newborn infants can learn word-like items from a short exposure and use this knowledge to generate predictions of following syllable succession. These results further suggest that predictive processing is innate, and it likely facilitates even the earliest recognition and learning of word forms. Revealing the role of predictive processing in early language development may have important implications for detecting and supporting infants at risk for language impairment.

## Funding

This project is conducted in co-operation with Hospital District of Helsinki and Uusimaa and supported by Research Funds of the 10.13039/100007797University of Helsinki (project Predictive coding in Language Learning, awarded to SY), the 10.13039/501100002341Academy of Finland (project no 316970), the National Research Fund of Hungary (grant number K115385 awarded to IW), and 10.13039/501100004756Emil Aaltonen Foundation (grant number 190235 awarded to ES).

## Declaration of Competing Interest

The authors declare that they have no known competing financial interests or personal relationships that could have appeared to influence the work reported in this paper.

## Data Availability

Data will be made available on request.

## Footnotes

1 Note that in infants, the ERP modulation elicited by deviants can appear with either polarity (for a review, see Kushnerenko et al., 2013); therefore, the infant equivalent of MMN is termed the mismatch response (MMR). MMR is useful measure for studying neonates because it can be recorded from sleeping infants (e.g., Alho et al., 1990).
