# Supplementary material for: More efficient formation of longer-term representations for word forms at birth can be linked to better language skills at 2 years
Source: Dev Cogn Neurosci. 2022 May 13;55:101113. doi: 10.1016/j.dcn.2022.101113 (PMC9130088; doi:10.1016/j.dcn.2022.101113)
Supplement: Supplementary file 1 — Supplementary material. [file mmc1.docx]

## Appendix A: Supplementary information


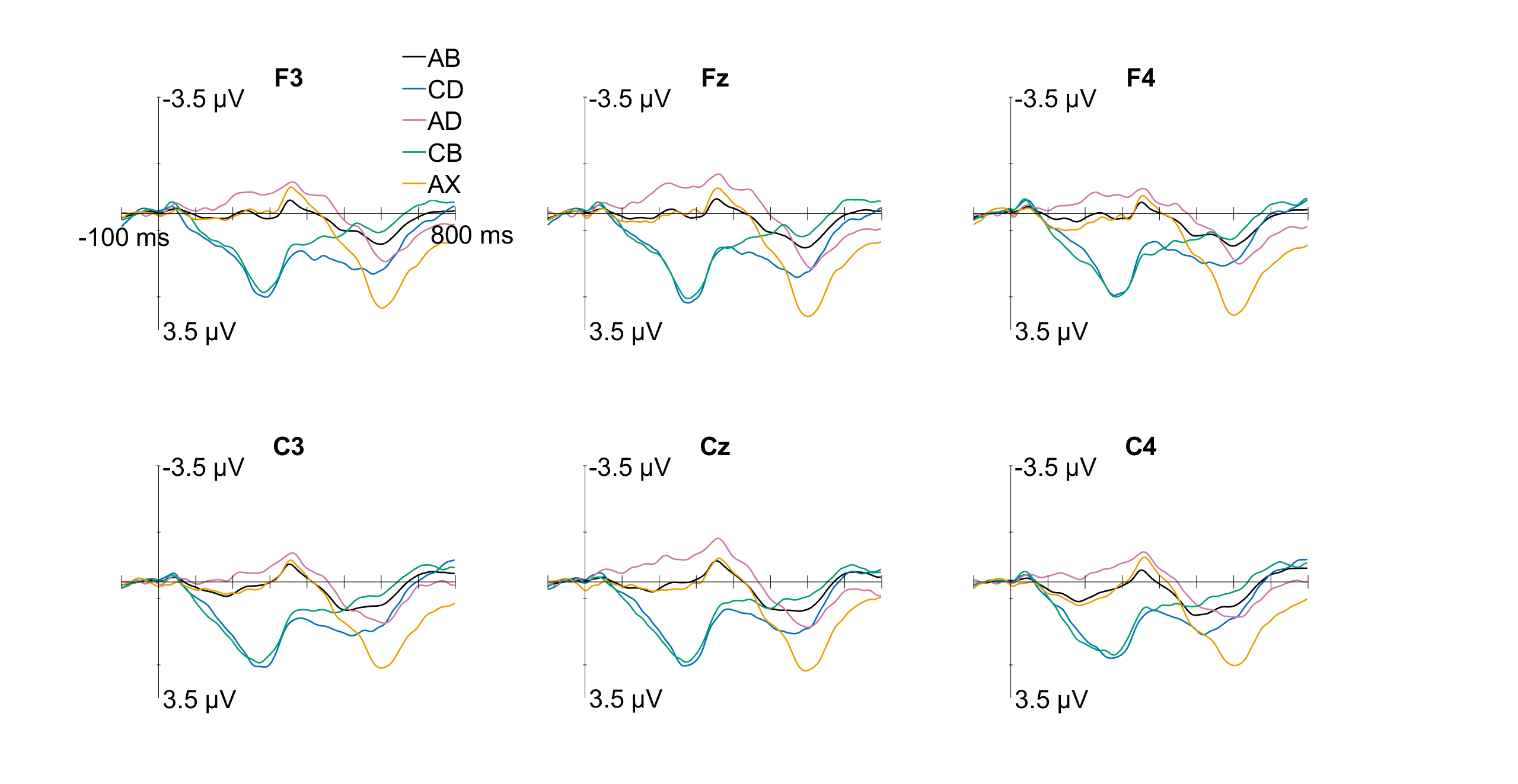


**Figure A.1** The group-averaged (N = 64) responses to the five (pseudo)words from the electrodes used in analyzes (*AB* - /kut:o/, the standard pseudoword; *CD* - /tek:ɑ/, the familiarized pseudoword; *AD* - /kuk:ɑ/, non-familiarized combination of two familiarized syllables; *CB* - /tet:o/, the other non-familiarized combination of two familiarized syllables *AX* - /kup:e/, a non-familiarized pseudoword starting out as the standard, but containing a syllable that was not familiarized). Note that here the baseline is -100 – 0 ms for all the (pseudo)words.

**Table A.1** ANOVA results from time window 1 (200 – 300 ms) for deviants with -100 – 0 ms baseline (*CD* & *CB*). In addition to the results discussed in the main text, there was a significant main effect of Laterality [F(2, 126) = 5.45, p < .01, ε = 0.905, η2 = 0.08] and significant Frontality × Laterality interaction [F(2, 126) = 4.24, p < .05, ε = 0.895, η2 = 0.06]. Bonferroni-corrected pairwise comparisons showed that the response was significantly more positive at the middle than at the right at the frontal electrodes [p < .05, d = 0.33], and significantly more negative at the right than at left or middle at the central electrodes [p < .05, d = 0.36, and p < 0.01, d = 0.47, respectively]. These effects describe the scalp distribution of the difference responses – see Figure 3.

| **Tests of Within-Subjects Effects** | | | | | | | |
| --- | --- | --- | --- | --- | --- | --- | --- |
| Measure: *CB* and *CD* minus *AB* amplitude (µV) from time window 1 | | | | | | | |
| Source | | Type III Sum of Squares | df | Mean Square | F | Sig. | Partial Eta Squared |
| Deviant | Sphericity Assumed | ,068 | 1 | ,068 | ,007 | ,936 | ,000 |
|  | Greenhouse-Geisser | ,068 | 1,000 | ,068 | ,007 | ,936 | ,000 |
|  | Huynh-Feldt | ,068 | 1,000 | ,068 | ,007 | ,936 | ,000 |
|  | Lower-bound | ,068 | 1,000 | ,068 | ,007 | ,936 | ,000 |
| Error(Deviant) | Sphericity Assumed | 649,236 | 63 | 10,305 |  |  |  |
|  | Greenhouse-Geisser | 649,236 | 63,000 | 10,305 |  |  |  |
|  | Huynh-Feldt | 649,236 | 63,000 | 10,305 |  |  |  |
|  | Lower-bound | 649,236 | 63,000 | 10,305 |  |  |  |
| Frontality | Sphericity Assumed | ,002 | 1 | ,002 | ,001 | ,978 | ,000 |
|  | Greenhouse-Geisser | ,002 | 1,000 | ,002 | ,001 | ,978 | ,000 |
|  | Huynh-Feldt | ,002 | 1,000 | ,002 | ,001 | ,978 | ,000 |
|  | Lower-bound | ,002 | 1,000 | ,002 | ,001 | ,978 | ,000 |
| Error(Frontality) | Sphericity Assumed | 133,213 | 63 | 2,114 |  |  |  |
|  | Greenhouse-Geisser | 133,213 | 63,000 | 2,114 |  |  |  |
|  | Huynh-Feldt | 133,213 | 63,000 | 2,114 |  |  |  |
|  | Lower-bound | 133,213 | 63,000 | 2,114 |  |  |  |
| Laterality | Sphericity Assumed | 9,762 | 2 | 4,881 | 5,452 | ,005 | ,080 |
|  | Greenhouse-Geisser | 9,762 | 1,810 | 5,392 | 5,452 | ,007 | ,080 |
|  | Huynh-Feldt | 9,762 | 1,861 | 5,246 | 5,452 | ,007 | ,080 |
|  | Lower-bound | 9,762 | 1,000 | 9,762 | 5,452 | ,023 | ,080 |
| Error(Laterality) | Sphericity Assumed | 112,800 | 126 | ,895 |  |  |  |
|  | Greenhouse-Geisser | 112,800 | 114,051 | ,989 |  |  |  |
|  | Huynh-Feldt | 112,800 | 117,230 | ,962 |  |  |  |
|  | Lower-bound | 112,800 | 63,000 | 1,790 |  |  |  |
| Deviant × Frontality | Sphericity Assumed | ,003 | 1 | ,003 | ,002 | ,962 | ,000 |
|  | Greenhouse-Geisser | ,003 | 1,000 | ,003 | ,002 | ,962 | ,000 |
|  | Huynh-Feldt | ,003 | 1,000 | ,003 | ,002 | ,962 | ,000 |
|  | Lower-bound | ,003 | 1,000 | ,003 | ,002 | ,962 | ,000 |
| Error(Deviant × Frontality) | Sphericity Assumed | 88,525 | 63 | 1,405 |  |  |  |
|  | Greenhouse-Geisser | 88,525 | 63,000 | 1,405 |  |  |  |
|  | Huynh-Feldt | 88,525 | 63,000 | 1,405 |  |  |  |
|  | Lower-bound | 88,525 | 63,000 | 1,405 |  |  |  |
| Deviant × Laterality | Sphericity Assumed | ,525 | 2 | ,262 | ,412 | ,663 | ,007 |
|  | Greenhouse-Geisser | ,525 | 1,983 | ,265 | ,412 | ,661 | ,007 |
|  | Huynh-Feldt | ,525 | 2,000 | ,262 | ,412 | ,663 | ,007 |
|  | Lower-bound | ,525 | 1,000 | ,525 | ,412 | ,523 | ,007 |
| Error(Deviant × Laterality) | Sphericity Assumed | 80,156 | 126 | ,636 |  |  |  |
|  | Greenhouse-Geisser | 80,156 | 124,917 | ,642 |  |  |  |
|  | Huynh-Feldt | 80,156 | 126,000 | ,636 |  |  |  |
|  | Lower-bound | 80,156 | 63,000 | 1,272 |  |  |  |
| Frontality × Laterality | Sphericity Assumed | 3,638 | 2 | 1,819 | 4,243 | ,016 | ,063 |
|  | Greenhouse-Geisser | 3,638 | 1,791 | 2,032 | 4,243 | ,020 | ,063 |
|  | Huynh-Feldt | 3,638 | 1,840 | 1,977 | 4,243 | ,019 | ,063 |
|  | Lower-bound | 3,638 | 1,000 | 3,638 | 4,243 | ,044 | ,063 |
| Error(Frontality × Laterality) | Sphericity Assumed | 54,014 | 126 | ,429 |  |  |  |
|  | Greenhouse-Geisser | 54,014 | 112,823 | ,479 |  |  |  |
|  | Huynh-Feldt | 54,014 | 115,909 | ,466 |  |  |  |
|  | Lower-bound | 54,014 | 63,000 | ,857 |  |  |  |
| Deviant × Frontality × Laterality | Sphericity Assumed | ,565 | 2 | ,282 | 1,031 | ,360 | ,016 |
|  | Greenhouse-Geisser | ,565 | 1,914 | ,295 | 1,031 | ,357 | ,016 |
|  | Huynh-Feldt | ,565 | 1,973 | ,286 | 1,031 | ,359 | ,016 |
|  | Lower-bound | ,565 | 1,000 | ,565 | 1,031 | ,314 | ,016 |
| Error(Deviant × Frontality × Laterality) | Sphericity Assumed | 34,487 | 126 | ,274 |  |  |  |
|  | Greenhouse-Geisser | 34,487 | 120,588 | ,286 |  |  |  |
|  | Huynh-Feldt | 34,487 | 124,277 | ,278 |  |  |  |
|  | Lower-bound | 34,487 | 63,000 | ,547 |  |  |  |

**Table A.2** ANOVA results from time window 2 (550 – 650 ms) for deviants with -100 – 0 ms baseline (*CD* & *CB*). No significant effects or interactions were found beyond the ones reported in the main text.

| **Tests of Within-Subjects Effects** | | | | | | | |
| --- | --- | --- | --- | --- | --- | --- | --- |
| Measure: *CB* and *CD* minus *AB* amplitude (µV) from time window 2 | | | | | | | |
| Source | | Type III Sum of Squares | df | Mean Square | F | Sig. | Partial Eta Squared |
| Deviant | Sphericity Assumed | 152,537 | 1 | 152,537 | 14,395 | ,000 | ,186 |
|  | Greenhouse-Geisser | 152,537 | 1,000 | 152,537 | 14,395 | ,000 | ,186 |
|  | Huynh-Feldt | 152,537 | 1,000 | 152,537 | 14,395 | ,000 | ,186 |
|  | Lower-bound | 152,537 | 1,000 | 152,537 | 14,395 | ,000 | ,186 |
| Error(Deviant) | Sphericity Assumed | 667,604 | 63 | 10,597 |  |  |  |
|  | Greenhouse-Geisser | 667,604 | 63,000 | 10,597 |  |  |  |
|  | Huynh-Feldt | 667,604 | 63,000 | 10,597 |  |  |  |
|  | Lower-bound | 667,604 | 63,000 | 10,597 |  |  |  |
| Frontality | Sphericity Assumed | 2,059 | 1 | 2,059 | 1,265 | ,265 | ,020 |
|  | Greenhouse-Geisser | 2,059 | 1,000 | 2,059 | 1,265 | ,265 | ,020 |
|  | Huynh-Feldt | 2,059 | 1,000 | 2,059 | 1,265 | ,265 | ,020 |
|  | Lower-bound | 2,059 | 1,000 | 2,059 | 1,265 | ,265 | ,020 |
| Error(Frontality) | Sphericity Assumed | 102,575 | 63 | 1,628 |  |  |  |
|  | Greenhouse-Geisser | 102,575 | 63,000 | 1,628 |  |  |  |
|  | Huynh-Feldt | 102,575 | 63,000 | 1,628 |  |  |  |
|  | Lower-bound | 102,575 | 63,000 | 1,628 |  |  |  |
| Laterality | Sphericity Assumed | 2,744 | 2 | 1,372 | 1,138 | ,324 | ,018 |
|  | Greenhouse-Geisser | 2,744 | 1,825 | 1,504 | 1,138 | ,320 | ,018 |
|  | Huynh-Feldt | 2,744 | 1,877 | 1,462 | 1,138 | ,321 | ,018 |
|  | Lower-bound | 2,744 | 1,000 | 2,744 | 1,138 | ,290 | ,018 |
| Error(Laterality) | Sphericity Assumed | 151,951 | 126 | 1,206 |  |  |  |
|  | Greenhouse-Geisser | 151,951 | 114,971 | 1,322 |  |  |  |
|  | Huynh-Feldt | 151,951 | 118,221 | 1,285 |  |  |  |
|  | Lower-bound | 151,951 | 63,000 | 2,412 |  |  |  |
| Deviant × Frontality | Sphericity Assumed | 1,179 | 1 | 1,179 | 1,161 | ,285 | ,018 |
|  | Greenhouse-Geisser | 1,179 | 1,000 | 1,179 | 1,161 | ,285 | ,018 |
|  | Huynh-Feldt | 1,179 | 1,000 | 1,179 | 1,161 | ,285 | ,018 |
|  | Lower-bound | 1,179 | 1,000 | 1,179 | 1,161 | ,285 | ,018 |
| Error(Deviant × Frontality) | Sphericity Assumed | 63,976 | 63 | 1,015 |  |  |  |
|  | Greenhouse-Geisser | 63,976 | 63,000 | 1,015 |  |  |  |
|  | Huynh-Feldt | 63,976 | 63,000 | 1,015 |  |  |  |
|  | Lower-bound | 63,976 | 63,000 | 1,015 |  |  |  |
| Deviant × Laterality | Sphericity Assumed | 2,830 | 2 | 1,415 | 1,884 | ,156 | ,029 |
|  | Greenhouse-Geisser | 2,830 | 1,946 | 1,455 | 1,884 | ,157 | ,029 |
|  | Huynh-Feldt | 2,830 | 2,000 | 1,415 | 1,884 | ,156 | ,029 |
|  | Lower-bound | 2,830 | 1,000 | 2,830 | 1,884 | ,175 | ,029 |
| Error(Deviant × Laterality) | Sphericity Assumed | 94,625 | 126 | ,751 |  |  |  |
|  | Greenhouse-Geisser | 94,625 | 122,570 | ,772 |  |  |  |
|  | Huynh-Feldt | 94,625 | 126,000 | ,751 |  |  |  |
|  | Lower-bound | 94,625 | 63,000 | 1,502 |  |  |  |
| Frontality × Laterality | Sphericity Assumed | ,059 | 2 | ,029 | ,075 | ,928 | ,001 |
|  | Greenhouse-Geisser | ,059 | 1,817 | ,032 | ,075 | ,913 | ,001 |
|  | Huynh-Feldt | ,059 | 1,868 | ,031 | ,075 | ,918 | ,001 |
|  | Lower-bound | ,059 | 1,000 | ,059 | ,075 | ,786 | ,001 |
| Error(Frontality × Laterality) | Sphericity Assumed | 49,512 | 126 | ,393 |  |  |  |
|  | Greenhouse-Geisser | 49,512 | 114,463 | ,433 |  |  |  |
|  | Huynh-Feldt | 49,512 | 117,674 | ,421 |  |  |  |
|  | Lower-bound | 49,512 | 63,000 | ,786 |  |  |  |
| Deviant × Frontality × Laterality | Sphericity Assumed | ,921 | 2 | ,460 | 1,602 | ,206 | ,025 |
|  | Greenhouse-Geisser | ,921 | 1,656 | ,556 | 1,602 | ,210 | ,025 |
|  | Huynh-Feldt | ,921 | 1,695 | ,543 | 1,602 | ,209 | ,025 |
|  | Lower-bound | ,921 | 1,000 | ,921 | 1,602 | ,210 | ,025 |
| Error(Deviant × Frontality × Laterality) | Sphericity Assumed | 36,219 | 126 | ,287 |  |  |  |
|  | Greenhouse-Geisser | 36,219 | 104,305 | ,347 |  |  |  |
|  | Huynh-Feldt | 36,219 | 106,766 | ,339 |  |  |  |
|  | Lower-bound | 36,219 | 63,000 | ,575 |  |  |  |

**Table A.3** Full ANOVA results from time window 2 (550 – 650 ms) for deviants with 0 – 300 ms baseline (*AD* & *AX*). No significant effects or interactions were found beyond the ones reported in the main text.

| **Tests of Within-Subjects Effects** | | | | | | | |
| --- | --- | --- | --- | --- | --- | --- | --- |
| Measure: *AD* and *AX* minus *AB* amplitude (µV) from time window 2 | | | | | | | |
| Source | | Type III Sum of Squares | df | Mean Square | F | Sig. | Partial Eta Squared |
| Deviant | Sphericity Assumed | 97,185 | 1 | 97,185 | 5,984 | ,017 | ,087 |
|  | Greenhouse-Geisser | 97,185 | 1,000 | 97,185 | 5,984 | ,017 | ,087 |
|  | Huynh-Feldt | 97,185 | 1,000 | 97,185 | 5,984 | ,017 | ,087 |
|  | Lower-bound | 97,185 | 1,000 | 97,185 | 5,984 | ,017 | ,087 |
| Error(Deviant) | Sphericity Assumed | 1023,223 | 63 | 16,242 |  |  |  |
|  | Greenhouse-Geisser | 1023,223 | 63,000 | 16,242 |  |  |  |
|  | Huynh-Feldt | 1023,223 | 63,000 | 16,242 |  |  |  |
|  | Lower-bound | 1023,223 | 63,000 | 16,242 |  |  |  |
| Frontality | Sphericity Assumed | 3,505 | 1 | 3,505 | 2,794 | ,100 | ,042 |
|  | Greenhouse-Geisser | 3,505 | 1,000 | 3,505 | 2,794 | ,100 | ,042 |
|  | Huynh-Feldt | 3,505 | 1,000 | 3,505 | 2,794 | ,100 | ,042 |
|  | Lower-bound | 3,505 | 1,000 | 3,505 | 2,794 | ,100 | ,042 |
| Error(Frontality) | Sphericity Assumed | 79,033 | 63 | 1,254 |  |  |  |
|  | Greenhouse-Geisser | 79,033 | 63,000 | 1,254 |  |  |  |
|  | Huynh-Feldt | 79,033 | 63,000 | 1,254 |  |  |  |
|  | Lower-bound | 79,033 | 63,000 | 1,254 |  |  |  |
| Laterality | Sphericity Assumed | 9,076 | 2 | 4,538 | 2,931 | ,057 | ,044 |
|  | Greenhouse-Geisser | 9,076 | 1,891 | 4,800 | 2,931 | ,060 | ,044 |
|  | Huynh-Feldt | 9,076 | 1,947 | 4,661 | 2,931 | ,058 | ,044 |
|  | Lower-bound | 9,076 | 1,000 | 9,076 | 2,931 | ,092 | ,044 |
| Error(Laterality) | Sphericity Assumed | 195,087 | 126 | 1,548 |  |  |  |
|  | Greenhouse-Geisser | 195,087 | 119,118 | 1,638 |  |  |  |
|  | Huynh-Feldt | 195,087 | 122,691 | 1,590 |  |  |  |
|  | Lower-bound | 195,087 | 63,000 | 3,097 |  |  |  |
| Deviant × Frontality | Sphericity Assumed | ,444 | 1 | ,444 | ,306 | ,582 | ,005 |
|  | Greenhouse-Geisser | ,444 | 1,000 | ,444 | ,306 | ,582 | ,005 |
|  | Huynh-Feldt | ,444 | 1,000 | ,444 | ,306 | ,582 | ,005 |
|  | Lower-bound | ,444 | 1,000 | ,444 | ,306 | ,582 | ,005 |
| Error(Deviant × Frontality) | Sphericity Assumed | 91,385 | 63 | 1,451 |  |  |  |
|  | Greenhouse-Geisser | 91,385 | 63,000 | 1,451 |  |  |  |
|  | Huynh-Feldt | 91,385 | 63,000 | 1,451 |  |  |  |
|  | Lower-bound | 91,385 | 63,000 | 1,451 |  |  |  |
| Deviant × Laterality | Sphericity Assumed | 4,340 | 2 | 2,170 | 1,471 | ,234 | ,023 |
|  | Greenhouse-Geisser | 4,340 | 1,775 | 2,446 | 1,471 | ,235 | ,023 |
|  | Huynh-Feldt | 4,340 | 1,822 | 2,382 | 1,471 | ,235 | ,023 |
|  | Lower-bound | 4,340 | 1,000 | 4,340 | 1,471 | ,230 | ,023 |
| Error(Deviant × Laterality) | Sphericity Assumed | 185,902 | 126 | 1,475 |  |  |  |
|  | Greenhouse-Geisser | 185,902 | 111,804 | 1,663 |  |  |  |
|  | Huynh-Feldt | 185,902 | 114,813 | 1,619 |  |  |  |
|  | Lower-bound | 185,902 | 63,000 | 2,951 |  |  |  |
| Frontality × Laterality | Sphericity Assumed | 8,605 | 2 | 4,302 | 3,382 | ,037 | ,051 |
|  | Greenhouse-Geisser | 8,605 | 1,380 | 6,234 | 3,382 | ,056 | ,051 |
|  | Huynh-Feldt | 8,605 | 1,401 | 6,141 | 3,382 | ,055 | ,051 |
|  | Lower-bound | 8,605 | 1,000 | 8,605 | 3,382 | ,071 | ,051 |
| Error(Frontality × Laterality) | Sphericity Assumed | 160,289 | 126 | 1,272 |  |  |  |
|  | Greenhouse-Geisser | 160,289 | 86,955 | 1,843 |  |  |  |
|  | Huynh-Feldt | 160,289 | 88,269 | 1,816 |  |  |  |
|  | Lower-bound | 160,289 | 63,000 | 2,544 |  |  |  |
| Deviant × Frontality × Laterality | Sphericity Assumed | 9,338 | 2 | 4,669 | 4,860 | ,009 | ,072 |
|  | Greenhouse-Geisser | 9,338 | 1,319 | 7,080 | 4,860 | ,021 | ,072 |
|  | Huynh-Feldt | 9,338 | 1,336 | 6,989 | 4,860 | ,021 | ,072 |
|  | Lower-bound | 9,338 | 1,000 | 9,338 | 4,860 | ,031 | ,072 |
| Error(Deviant × Frontality × Laterality) | Sphericity Assumed | 121,039 | 126 | ,961 |  |  |  |
|  | Greenhouse-Geisser | 121,039 | 83,091 | 1,457 |  |  |  |
|  | Huynh-Feldt | 121,039 | 84,172 | 1,438 |  |  |  |
|  | Lower-bound | 121,039 | 63,000 | 1,921 |  |  |  |

**Table A.4** Rank correlations between for the three contrasts between responses to pseudowords with identical first syllables representing the three potential regularity violation effects: longer-term representation (*CD* vs. *CB*), local context (*AD* vs. *AX*), and syllable novelty (*AD* vs. *AB*). Note that these correlations are not corrected for multiple comparisons.

**Correlations**

|  | | | *CD* vs. *CB* | *AD* vs. *AX* | *AD* vs. *AB* |
| --- | --- | --- | --- | --- | --- |
| Spearman's rho | *CD* vs *CB* | Correlation Coefficient | N/A | ,034 | ,199 |
|  |  | Sig. (2-tailed) | N/A | ,790 | ,116 |
|  |  | N | N/A | 64 | 64 |
|  | *AD* vs *AX* | Correlation Coefficient | ,034 | N/A | ,717^**^ |
|  |  | Sig. (2-tailed) | ,790 | N/A | ,000 |
|  |  | N | 64 | N/A | 64 |
|  | *AD* vs *AB* | Correlation Coefficient | ,199 | ,717^**^ | N/A |
|  |  | Sig. (2-tailed) | ,116 | ,000 | N/A |
|  |  | N | 64 | 64 | N/A |
| *. Correlation is significant at the 0.05 level (2-tailed). | | | | | |
| **. Correlation is significant at the 0.01 level (2-tailed). | | | | | |

**Table A.5** Rank correlations between MLU and the remaining regularity violation effects (local context, *AD* vs. *AX*; and syllable novelty, *AD* vs. *AB*). Note that these correlations are not corrected for multiple comparisons.

**Correlations**

|  | | | *AD* vs. *AX* | *AD* vs. *AB* |
| --- | --- | --- | --- | --- |
| Spearman's rho | MLU | Correlation Coefficient | -,146 | -,095 |
|  |  | Sig. (2-tailed) | ,337 | ,534 |
|  |  | N | 45 | 45 |
| *. Correlation is significant at the 0.05 level (2-tailed). | | | | |
| **. Correlation is significant at the 0.01 level (2-tailed). | | | | |

**Table A.6** Rank correlations between contrast representing longer-term representation (*CD* vs. *CB*), MLU, and the CDI vocabulary measure. Note that these correlations are not corrected for multiple comparisons.

|  | | | MLU | CDI vocabulary measure |  |
| --- | --- | --- | --- | --- | --- |
| Spearman's rho | CD vs. CB | Correlation Coefficient | ,334^*^ | ,211 |  |
|  |  | Sig. (2-tailed) | ,025 | ,164 |  |
|  |  | N | 45 | 45 |  |
| *. Correlation is significant at the 0.05 level (2-tailed). | | | | | |
| **. Correlation is significant at the 0.01 level (2-tailed). | | | | | |
